# Rh@Au Core–Shell Nanocrystals with the Core in Tensile Strain and the Shell in Compressive Strain

**DOI:** 10.1021/acs.jpcc.3c06793

**Published:** 2024-01-15

**Authors:** Veronica D. Pawlik, Annemieke Janssen, Yong Ding, Younan Xia

**Affiliations:** † School of Chemistry and Biochemistry, Georgia Institute of Technology, Atlanta, Georgia 30332, United States; ‡ School of Material Science and Engineering, Georgia Institute of Technology, Atlanta, Georgia 30332, United States; § The Wallace H. Coulter Department of Biomedical Engineering, Georgia Institute of Technology and Emory University, Atlanta, Georgia 30332, United States

## Abstract

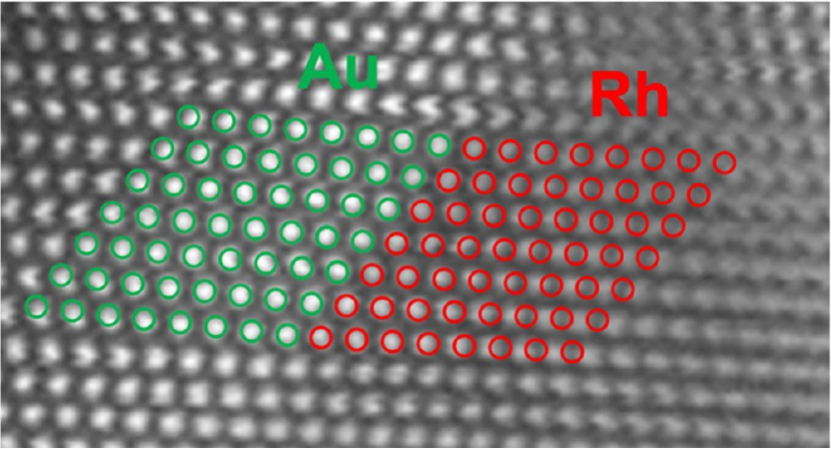

Bimetallic nanocrystals provide a versatile platform for utilizing the desired characteristics of two different elements within one particle. Core–shell nanocrystals, in particular, have found widespread use in catalysis by providing an ability to leverage the strains arising from the lattice mismatch between the core and the shell. However, large (>5%) lattice mismatch tends to result in nonepitaxial growth and lattice defects in an effort to release the strain. Herein, we report the epitaxial growth of Au on Rh cubic seeds under mild reaction conditions to generate Rh@Au truncated octahedra featuring a lattice mismatch of 7.2%. Key to the success was the use of small (4.5 nm) Rh cubes as seeds, which could homogeneously distribute the tensile strain arising from the epitaxial growth of a conformal, compressively strained Au shell. Further, delicate tuning of kinetic parameters through the introduction of NaOH and KBr into the synthesis allowed for a unique nucleation pattern that led to centrally located cores and a narrow size distribution for the product. A thorough investigation of the various possible highly strained morphologies was conducted to gain a full understanding of the system.

## Introduction

Bimetallic nanocrystals have greatly expanded the versatility of nanomaterials.^1^ Bringing together two metals with distinct properties has enabled new applications in catalysis, plasmonics, and surface-enhanced Raman spectroscopy.^2−4^ These nanocrystals can be broadly divided into three categories based on the spatial distributions of constituent elements: core–shell, heterostructured, alloy, or intermetallic. Each configuration has unique characteristics and advantages, as exemplified by the increased thermal stability or novel optical properties in core–shelled, tandem catalysis in heterostructured, and synergistic effects in alloyed nanocrystals.^5−8^ Among different activities, enhancement of catalytic activity through the surface strain associated with core–shell nanocrystals remains one of the most exploited advantages of bimetallic nanocrystals.^9−12^ The presence of strain in the shell region invariably results in surface reconstruction and thereby shifts in the *d*-band center.^13^ This shift, in turn, alters the adsorption and desorption energies of reactants and/or intermediates, affecting the catalytic activity and/or selectivity. Yet, it is this very feature that has imposed a major limitation on the synthesis of core–shell nanocrystals.^14^

A prior study of Pd and Ag growth on Au by Fan et al. set the upper limit for lattice mismatch at 5% for epitaxial growth.^15^ Although a number of core–shell nanocrystals with higher lattice mismatches have since been synthesized,^16−19^ heterostructures with discontinuous coatings on the surface are much more common.^20−23^ Among different metals, Cu has become a popular choice as the coating material, with examples of core–shell nanocrystals being derived from both Pd and Au seeds corresponding to lattice mismatches of 7.1 and 11.4%, respectively.^16−18^ One of the first examples of bimetallic core–shell nanocrystals with a lattice mismatch above 5% was the synthesis of various Au@Cu polygons.^19^ Specifically, Tsuji et al. used a polyol synthesis under microwave heating to produce Au polygonal nanocrystals, including octahedra, nanoplates, decahedra, and icosahedra, and then applied them as seeds for Cu deposition. It is worth noting that all of the seeds were enclosed by {111} facets. Further analysis of the growth mode revealed that the large lattice mismatch impaired growth along the corners and edges. Thus, particles with large facets and few corners and edges, such as octahedra and nanoplates, preserved the {111} facets well. However, particles with many corners and edges, such as icosahedra, lost this facet definition. Another report of significantly mismatched Cu core–shell nanocrystals was grown on Pd cubic seeds.^16^ In this case, Jin et al. grew Cu on Pd nanocubes in the presence of hexadecylamine (HDA) and obtained Pd@Cu core–shell nanocubes. Similar to the report by Tsuji et al., epitaxial growth of Cu was impaired at corners and edges. Instead, Cu nucleated on flat side faces before moving across the Pd surface to coat the entire particle. This process also resulted in cores that were displaced from the center of the final nanocrystal. When the Pd cubic seeds were replaced with either cuboctahedra or octahedra, the resulting core–shell nanocrystals still formed nanocubes with well-defined {100} facets. The shape preference can be attributed to HDA, which is a selective capping agent for Cu{100} facets. Subsequent reports by both Lyu et al. and Hsia et al. once again grew Cu on Au seeds.^17,18^ In these studies, HDA was used as a shape-directing agent to generate Au@Cu nanocubes from small Au nanospheres as well as both Au@Cu nanocubes and octahedra from Au octahedral seeds.

In all of these syntheses, the large lattice mismatch was explored under tensile strain, meaning that the native lattice spacing for the shell material is smaller than that for the core. Examples of compressive strain in bimetallic core–shell structures are rarer. An example of multishelled Pd@Au nanocrystals with alternating Pd and Au layers was reported by Wang et al.^24^ However, the lattice mismatch between Pd and Au is only 4.8%, falling under the originally established 5% limit. Likewise, the work by Gamler et al. explored the distributions of strain in three examples of core–shell nanoparticles with a maximum compressive strain of 3.1%.^25^ In this work, we chose Rh and Au to examine the influence of the compressive strain on a highly mismatched system. Specifically, we demonstrated the successful synthesis of 13.8 ± 1.1 nm single-crystal Rh@Au nanocrystals using a simple, room-temperature, aqueous protocol that involved the use of ascorbic acid (AA) as a reducing agent. Careful kinetic control through dropwise injection enabled heterogeneous nucleation of Au on the Rh cubic seeds. Our results suggested that the core–shell nanocrystals were formed through localized, epitaxial growth of Au on Rh facets, in line with previous literature. The Rh@Au nanocrystals evolved into truncated octahedra largely covered by {111} facets. Without the presence of any shape-directing agents, the nanocrystals naturally took on a thermodynamic morphology with the lowest surface energy. We believe that the principles of lattice-mismatched epitaxial growth discussed in this work can be extended beyond the Rh@Au system. While the specific reagents used in this study might not translate across systems due to differences in reduction potential, solubility, and precursor, the fundamental principles of tuning deposition vs. diffusion, do.

## Experimental Section

### Chemicals and Materials

Poly(vinylpyrrolidone) (PVP, 55,000 in molecular weight), l-ascorbic acid (AA, 99.0%), sodium hydroxide (NaOH, 97%), potassium bromide (KBr, 99.0%), tetrachloroauric(III) acid trihydrate (HAuCl_4_·3H_2_O, 99%), and sodium hexachlororhodate(III) (Na_3_RhCl_6_, 99%) were all obtained from Sigma-Aldrich. Ethylene glycol (EG, 99%) was purchased from J. T. Baker. All aqueous solutions were prepared using deionized water with a resistivity of 18.2 MΩ·cm at room temperature.

### Characterizations

All transmission electron microscopy (TEM) images were captured using a Hitachi HT7700 microscope at 120 kV. The sample was prepared by adding a drop of colloidal suspension onto a carbon-coated Cu grid, followed by drying at ambient temperature. Scanning TEM (STEM) images and energy-dispersive X-ray spectroscopy (EDX) elemental mapping were taken on a Hitachi HD-2700 microscope at 200 kV equipped with a Bruker SSD EDX detector.

### Synthesis of the 4.5 nm Rh Cubic Seeds

The Rh seeds were synthesized using a previously published protocol.^26^ Briefly, 133 mg of PVP, 52.8 mg of AA, and 108 mg of KBr were dissolved in 13 mL of EG in a 150 mL round-bottom glass flask. The solution was then preheated under magnetic stirring at 140 °C for 1 h in an oil bath. Simultaneously, Na_3_RhCl_6_ was dissolved in 6 mL of EG to obtain a solution with a concentration of 7.7 mg/mL. Once the reaction solution had finished preheating, 1.1 mL of the precursor solution was injected at 60 mL/h, immediately followed by the remaining 4.9 at 4 mL/h. The reaction was allowed to proceed for a total of 3 h at 140 °C. Once the reaction was complete, the solution was cooled to room temperature before the particles were collected and washed. In the first round, the particles were crashed out with acetone at a 1:3 volume ratio and centrifuged at 12,000 rpm for 45 min. The particles were then dispersed in a 1:3 (vol) ethanol/acetone mixture and centrifuged twice more at 17,500 rpm for 20 min. Finally, the particles were redispersed in water.

### Synthesis of Rh@Au Nanoparticles

In a typical synthesis, 10 mg of PVP and 5 mg of AA were dissolved in 3 mL of water in a 20 mL glass vial. Then, 30 μL of 3.125 M NaOH, 15 μL of 40 mM KBr, and 20 μL of the washed Rh seeds were added to the solution and placed under magnetic stirring at room temperature (22 °C). Finally, 1.5 mL of 0.28 mM HAuCl_4_ was injected at 0.200 mL/h. After injection was complete, the particles were collected by centrifugation at 17,500 rpm for 10 min and then redispersed in water. This washing procedure was repeated three times.

## Results and Discussion

The synthesis of Rh@Au core–shell nanocrystals was separated into two steps. In the first step, single-crystal Rh nanocubes with an average edge length of 4.5 nm were synthesized using a protocol previously reported by our group.^26^ Specifically, the nanocubes were prepared by using a one-pot approach divided into two distinct kinetic regimes. For the nucleation step, Na_3_RhCl_6_ was rapidly injected into an ethylene glycol (EG) solution containing poly(vinylpyrrolidone) (PVP), AA, and KBr. This was immediately followed by a slower injection of the precursor to promote the formation of a cubic shape with the help of KBr. The Rh nanocubes were washed and then redispersed in water for subsequent growth. Figure S1 shows a transmission electron microscopy (TEM) image as well as a high-angle annular dark-field scanning TEM (HAADF-STEM) image of the as-synthesized Rh nanocubes.

**Figure 1:**
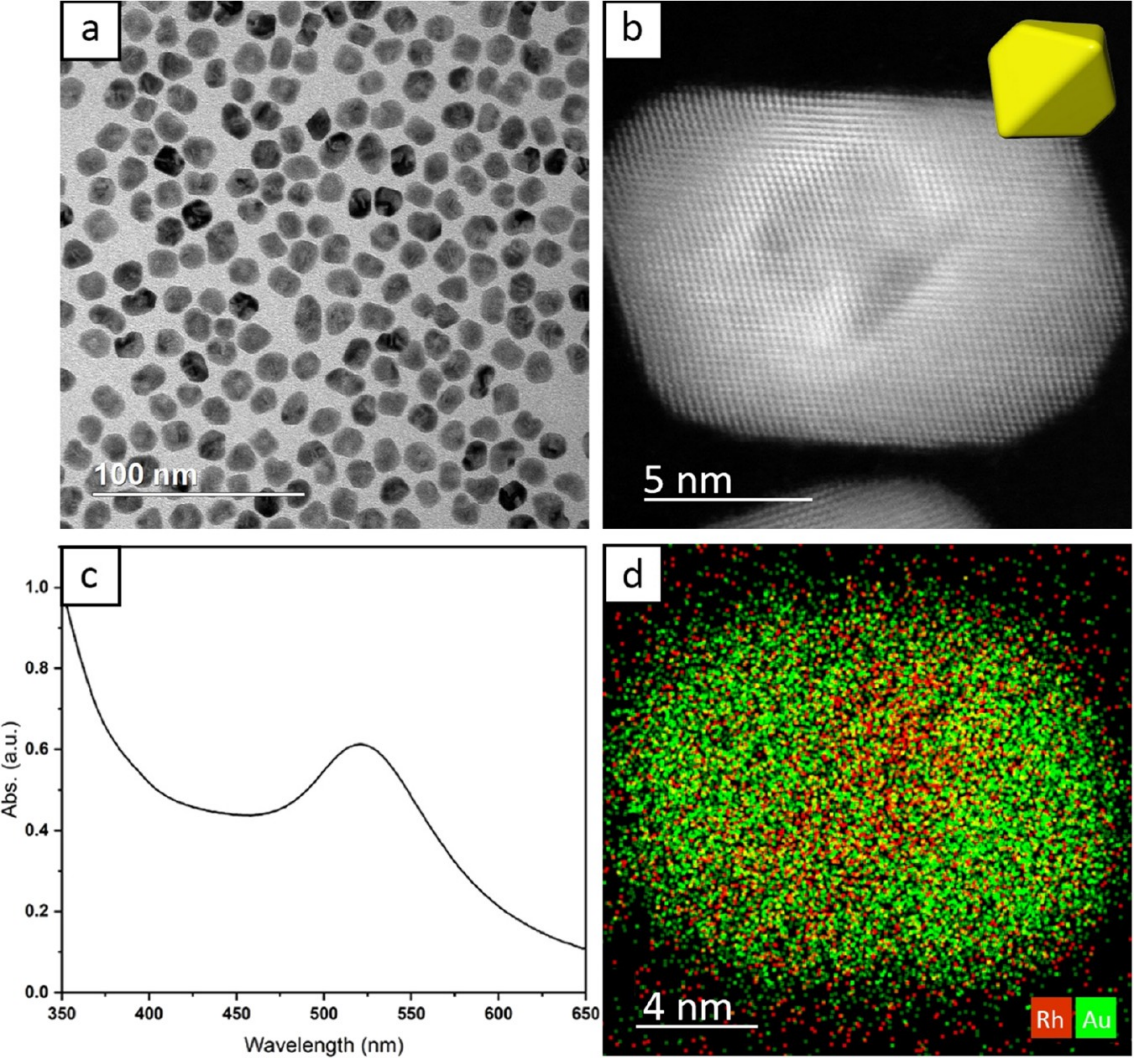
(a) TEM image of the Rh@Au nanocrystals. (b) HAADF-STEM image of a Rh@Au nanocrystal with the inset showing a model in the same orientation. (c) UV–vis spectrum recorded from an aqueous suspension of the nanocrystals. (d) EDX mapping corresponding to the STEM image.

Once washed, the Rh nanocubes were added to an aqueous solution containing PVP, AA, NaOH, and KBr. Aqueous HAuCl_4_ was then injected at a controlled rate to produce Rh@Au core–shell nanocrystals. Figure 1a shows a typical TEM image of the products obtained by using the standard protocol. The particles were predominantly single crystals (>80%) and exhibited a truncated octahedral shape (>75%), with a Rh seed embedded inside. The core–shell structure is supported by both a slightly lighter color of the Rh seed due to the difference in atomic number, as well as the obvious Moiré pattern produced by the mismatch in lattice spacing. These moiré patterns alongside strain can cause irregular contrast, giving the initial impression of a higher proportion of twinned nanocrystals. However, careful analysis of the nanocrystals confirmed that they were predominantly single crystals.^16^ The HAADF-STEM image in Figure 1b further demonstrates this structure and confirms the single-crystal nature of the particle, with no faults apparent at the Rh–Au interface. The inset in Figure 1b shows a model of the truncated octahedron in the same orientation. Figure 1c shows a typical UV–vis spectrum recorded from an aqueous suspension of the Rh@Au octahedra with a single peak at 521 nm, in agreement with Au quasi-spherical particles of a similar size.^27^ Finally, Figure 1d provides an energy-dispersive X-ray spectroscopy (EDX) elemental mapping of the Rh@Au truncated octahedron. The distributions of both Rh (red) and Au (green) show a difference in composition between the core and the shell that is in line with the contrast difference observed in Figure 1b, confirming again the bimetallic core–shell structure of the nanocrystal. An additional EDX map of another Rh@Au nanocrystal with a corresponding line scan can be found in the Supporting Information (Figure S2).

**Figure 2:**
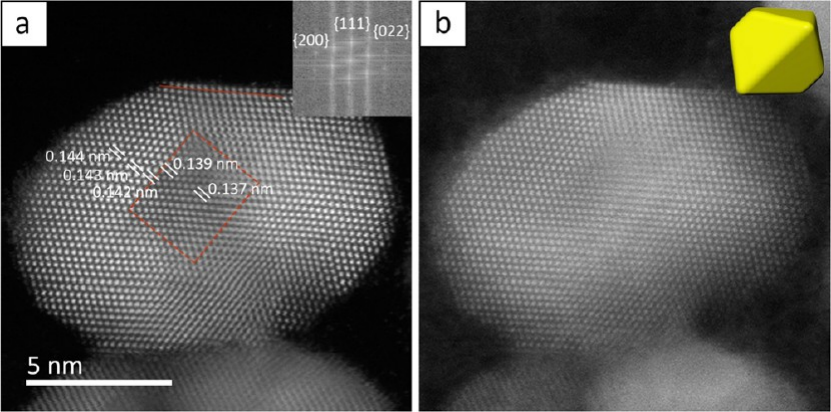
(a) HAADF-STEM image of a core–shell truncated octahedron with the corresponding FFT in the inset. The red rectangle outlines the profile of the Rh seed and the red line across the top emphasizes the distortion in the Au lattice. Lattice spacings along the (022) reflection are labeled at various distances from the Rh/Au interface. (b) SE-STEM image of the same particle, with the inset showing a model of the truncated octahedron oriented in the same direction. The scale bar applies to both panels.

As discussed in the Introduction section, the limitation of epitaxial growth resulting in well-faceted core–shell nanocrystals has been set at a lattice mismatch of under 5%.^15^ Despite this, examples of well-faceted, single-crystal core–shell nanocrystals with lattice mismatches as high as 11.4% have been reported.^18^ Traditionally, in lattice-mismatched nanocrystals, deformation and strain are expected and indeed observed in the shell.^10^ Assuming full coverage of the core region, such an emphasized focus on the shell, especially in the context of catalysis, is warranted. Furthermore, the large sizes of the cores (e.g., 18 nm Pd cubes) typically employed for the core–shell syntheses minimize the effects of strain on the core because strain decays exponentially perpendicular to the core–shell interface.^16,28^ This means that only the first few atomic layers are affected, making up a small percentage of the overall core volume. The bimetallic Rh@Au octahedra synthesized in this work are somewhat unique in this respect because the Rh cores are significantly smaller. The only other example of large lattice mismatch in a bimetallic system with a core of comparable size is the synthesis reported by Lyu et al.; however, strain effects on the core were not explicitly mentioned.^17^ A study on lattice mismatch in semiconductors has indicated that in the conventional regime of tensile strain, a small core can more effectively distribute the strain over its volume to accommodate epitaxial growth of a defect-free shell.^29^ The atomic-resolution HAADF-STEM images in Figure 2 suggest that a similar mechanism is also present under compressive strain.

Figure 2a shows a HAADF-STEM image of a typical Rh@Au octahedron taken along the [110] direction, as indicated by the fast Fourier Transform (FFT) in the inset. The core region is indicated by a dashed red outline, and the *d* spacings of the (022) reflection are provided at various locations with respect to the core. The lattice constants for Au and Rh are 0.408 and 0.380 nm, which correspond to *d*_*022*_ spacings of 0.144 and 0.134 nm, respectively. Deviations from the calculated values are predictably most pronounced at the Rh–Au interface with Au compressed to 0.142 nm and Rh expanded to 0.139 nm. The Au lattice fully relaxed to a *d*_*022*_ spacing of 0.144 nm after eight atomic layers. However, the *d* spacing at an equivalent eight atomic layers measured at the center of the Rh seed only relaxed to 0.137 nm. This result indicates that the thick epitaxial Au shell was able to expand the Rh lattice over nearly the entire volume of the seed to compensate for the large lattice mismatch. Despite significant strain relaxation in Au by the eighth atomic layer, long-range crystal distortion still occurred in the Au shell. The straight red line near the top of the nanocrystal in Figure 2a serves as a visual reference to illustrate compression of the lattice in areas closer to the core. Figure 2b shows an atomically resolved secondary electron STEM (SE-STEM) image that clearly depicts the {111} facets of the Rh@Au octahedron. The inset shows a corresponding model of a truncated octahedron oriented in the same direction. In addition to the electron microscopy analysis, X-ray diffraction (XRD) patterns were collected to provide more information about the strain (Figure S3). The peaks corresponding to Au were all shifted to slightly higher angles, indicating contraction of the lattice, while the Rh peaks were shifted to lower angles, corresponding to an expansion of the lattice. The Rh peaks for both the Rh seeds and Rh@Au nanocrystals appeared at lower angles than the reference peaks due to the ultrasmall sizes of the particles. The peaks of the Rh@Au nanocrystals were shifted more, indicating a larger *d* spacing.

**Figure 3:**
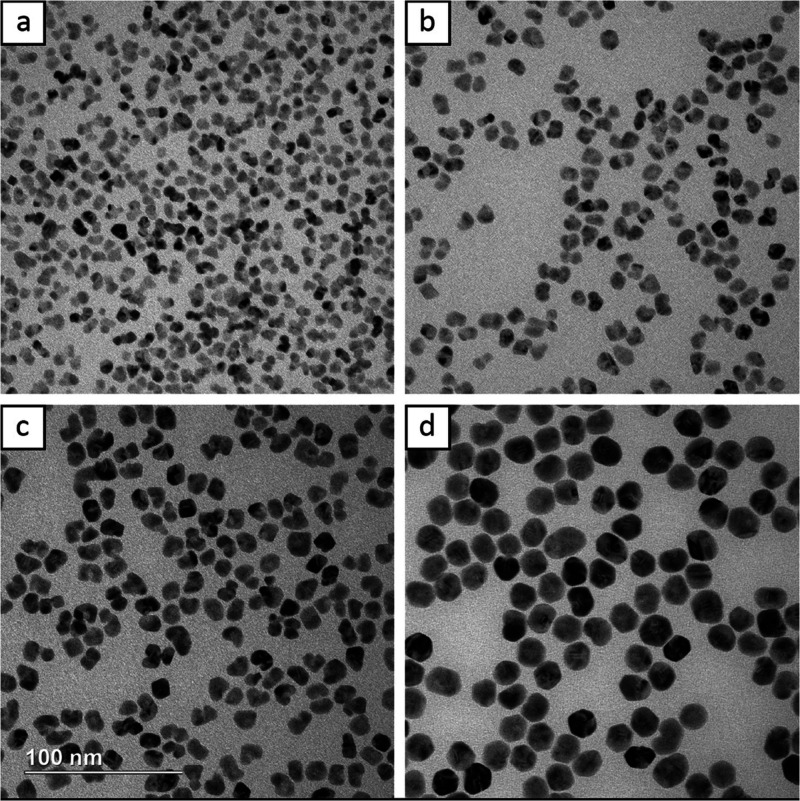
TEM images of particles during various phases of growth after (a) 0.25, (b) 0.50, (c) 1.00, and (d) 3.00 mL of the HAuCl_4_ precursor had been injected. Final concentrations of the HAuCl_4_ precursor were (a) 0.020, (b) 0.040, (c) 0.070, and (d) 0.138 mM. The scale bar applies to all panels.

To better understand the formation of the single crystal Rh@Au octahedra, we collected samples after various volumes of the Au(III) precursor were injected during the standard protocol. Figure 3a–c shows TEM images of samples after 0.25, 0.50, and 1.00 mL of the precursor had been injected, respectively. These correspond to final concentrations of 0.020, 0.040, and 0.070 mM of the Au precursor. Similar to previous studies, Figure 3a shows that the Au atoms preferentially nucleated on the faces of the Rh seeds instead of the corners and edges higher in energy.^19^ However, the Au atoms also tended to nucleate simultaneously on two opposite faces instead of nucleating on either one or two adjacent faces.^16^ These two Au islands subsequently served as the preferential sites for further Au deposition, as depicted in Figure 3b,c. This mode of nucleation explains why the final product results in a truncated octahedron. Understanding this growth mechanism and looking at Figure 1b, it can be seen that the two original Au nucleation points were the top left and bottom right of the seed, where thicker Au overlayers were located. This symmetrical growth also explains the more or less central location of the Rh core in the final octahedron as well as the progressively increasing proportion of octahedral nanocrystals. Figure 3d shows the nanocrystals after 3.00 mL (0.138 mM) of the precursor had been injected. The products were significantly more rounded with much of the asymmetry (both “kidney bean” and elongation) eliminated. This suggests that once the Rh seeds were fully coated, Au nucleated more evenly across the entire surface, shifting from island growth mode to layer-by-layer growth. Figure S4 shows the corresponding UV–vis spectra, which indicate a slight red-shift of the peak as the nanocrystals increased in size.

**Figure 4:**
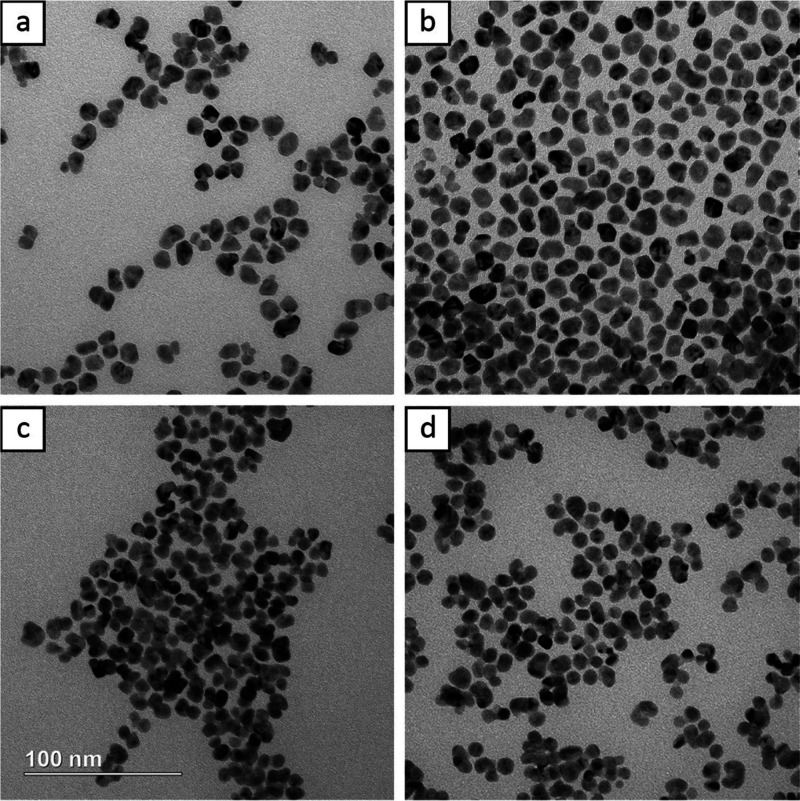
TEM images of nanocrystals synthesized at injection rates of (a) 0.050, (b) 0.100, (c) 0.300, and (d) one-shot. The scale bar applies to all panels.

Deposition and diffusion are strongly tied to the reaction kinetics. Figure 4 shows TEM images of Rh@Au nanocrystals synthesized at different precursor injection rates. Controlling kinetics by using slow injection rates in a seed-mediated synthesis is known to induce symmetry breaking.^20^ The effect is exaggerated in lattice-mismatched systems because of the already high barrier to surface diffusion. Figure 4a,b depict nanocrystals synthesized at injection rates of 0.050 and 0.100 mL/h, respectively. Using a 0.050 mL/h injection rate promoted more dumbbell-shaped particles with two Au islands on opposite faces. As the rate increased to 0.100 mL/h, injection was fast enough to significantly promote merging along one side between the two Au islands, creating more kidney bean-shaped nanocrystals, leaving one face of the Rh seed exposed. At 0.300 mL/h, nanocrystals with three distinct Au islands began to appear (Figure 4c). In this case, the concentration of Au(III) precursor in solution was high enough to induce more nucleation sites but the deposition rate also outpaced the rate of diffusion, leading to distinct Au islands. Finally, when one shot injection was used, the products became irregular, with an observable amount of self-nucleation. This can be seen by the appearance of some icosahedra and other twinned particles alongside the mix of Rh–Au particles (Figure 4d). This is consistent with Figure S5, which shows that icosahedra are the predominant self-nucleation product when the standard protocol was run in the absence of Rh seeds.

**Figure 5:**
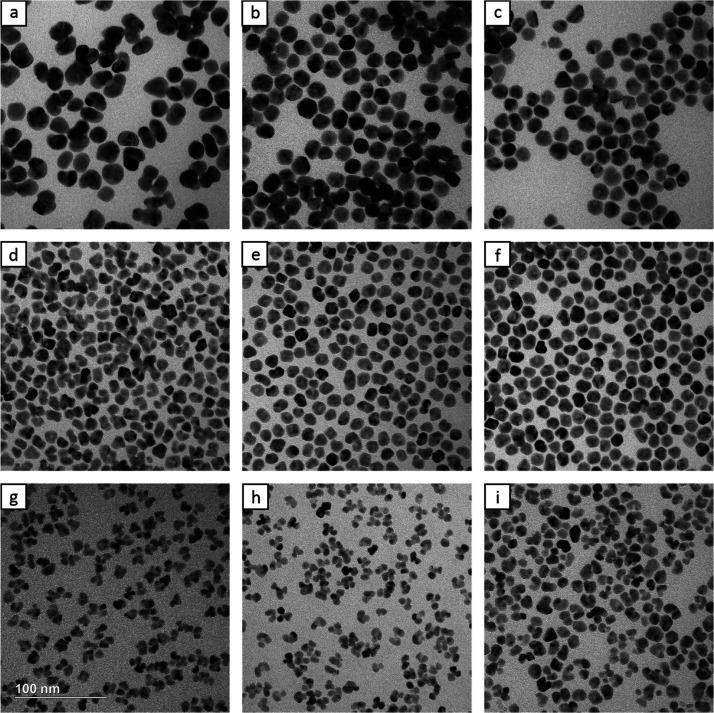
(a–i) TEM images of Rh@Au nanocrystals synthesized with different amounts of 3.125 M NaOH and 40 mM KBr. The volume of NaOH added increases from 0 to 30 and 50 μL descending by row. These correspond to final concentrations of approximately 0, 20.55, and 34.10 mM NaOH, respectively. The volume of KBr added increases from 0 to 15 and 20 μL moving right by column. These correspond to final concentrations of approximately 0, 0.13, and 0.17 mM KBr, respectively. The scale bar applies to all panels.

The reaction kinetics can be more delicately tuned through the addition of KBr and/or NaOH. The Br^–^ ion is often used as a capping agent for the {100} facets on metals.^30^ In the present work, it can also serve as a kinetic knob through ligand exchange with the HAuCl_4_ precursor. Because AuBr_4_^–^ has a lower reduction potential than AuCl_4_^–^, increasing the amount of KBr will increase the concentration of AuBr_4_^–^ and slow down the reaction kinetics.^31^ Adding NaOH has the opposite effect as AA can exist in three distinct forms depending on pH.^32^ By increasing the pH through the addition of NaOH, AA can exist in either the ascorbate or the diascorbate form, both of which have a higher reducing power. Thus, carefully balancing the relative concentrations of both KBr and NaOH significantly impacts the final particle morphology. Figure 5 shows the products obtained by varying the concentrations of both KBr and NaOH. The volume of NaOH added increases from 0 to 30 and 50 μL, descending by row. Looking at the center column, the TEM image in Figure 5b shows irregular Rh@Au core–shell nanocrystals. Because no NaOH was present in the synthesis, AA was in its weakest form and thus the slowest reduction rate. The biggest consequence of this was decreasing the number of nucleation sites below two per seed. This led to the production of Rh@Au nanocrystals with displaced cores that were more difficult to resolve, unless the particle was oriented advantageously. The larger particle size can also be understood to be a consequence of Rh seeds with no nucleation sites that effectively increase the concentration of the Au(III) precursor. Moving down the column, the TEM image in Figure 5e shows the standard protocol where the kinetics are balanced to produce truncated octahedra. The double nucleation sites, as discussed previously, produced more symmetrical nanocrystals with centrally located seeds. Figure 5h corresponds to the case with an excess amount of NaOH, which significantly increased the reducing power of AA to promote multiple nucleation sites, similar to the increased injection rate in Figure 4c.

**Figure 6:**
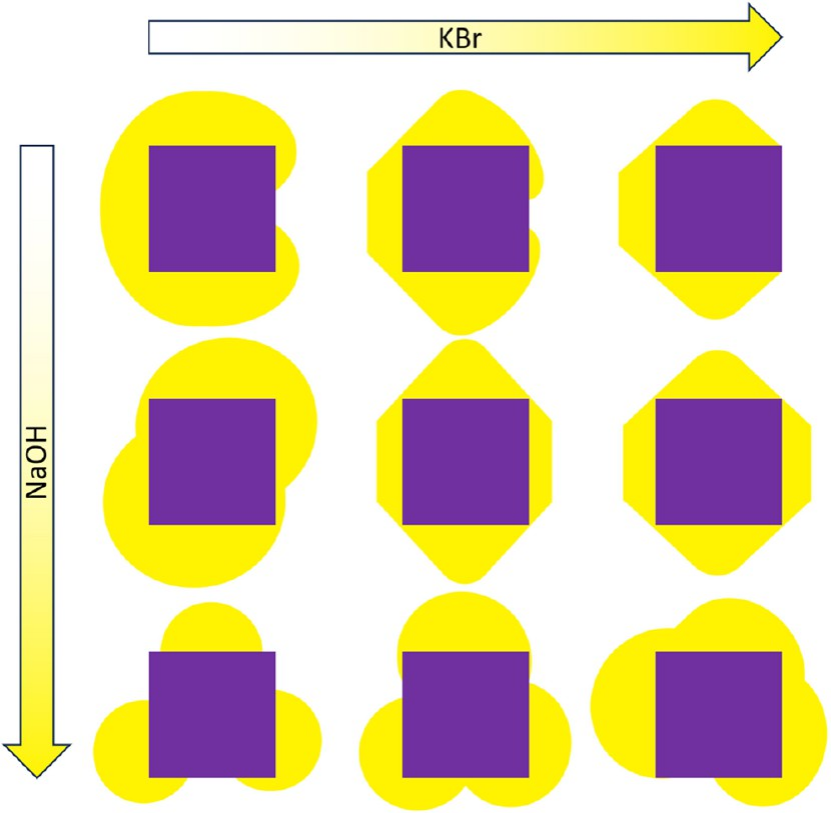
Schematic depiction of the kinetic effects that the addition of more NaOH or KBr has on crystal growth.

Analyzing Figure 5 from left to right, the concentration of KBr increased from 0 to 15 and 20 μL. Looking at the center row, Figure 5d shows Rh@Au nanocrystals with a unique morphology. While most of the Rh seeds were fully enclosed, the two Au islands were still easily discernible, forming bulges and creating a bow-tie shape. This is because the reducing power of AA was increased enough to ensure two nucleation sites, while the lack of KBr shifted the balance even farther toward deposition over diffusion. Figure 5f involved an increased concentration of KBr. Because the reduction strength was still moderate from the addition of NaOH, full coverage could be achieved, and the KBr worked by promoting diffusion relative to reduction. This resulted in slightly more rounded nanocrystals similar to those described in Figure 3d. Both NaOH and KBr worked by controlling the reduction rate, running directly counter to each other. However, their relative strengths and modes of action suggest that instead of canceling out, they could each be used to achieve distinct kinetic parameters. Since NaOH controlled the number of nucleation sites, the first row exhibits 0–1, the middle 2, and the bottom 3. Meanwhile, the addition of KBr slowed the reduction, allowing more Au diffusion relative to Au deposition. This resulted in nanocrystals with clear Au bulges in the left column morphing to more rounded nanocrystals in the right column. The trends are summarized in the schematic depiction in Figure 6. Although not explicitly described, Figure 5a,c,g,i also follows these trends.

**Figure 7:**
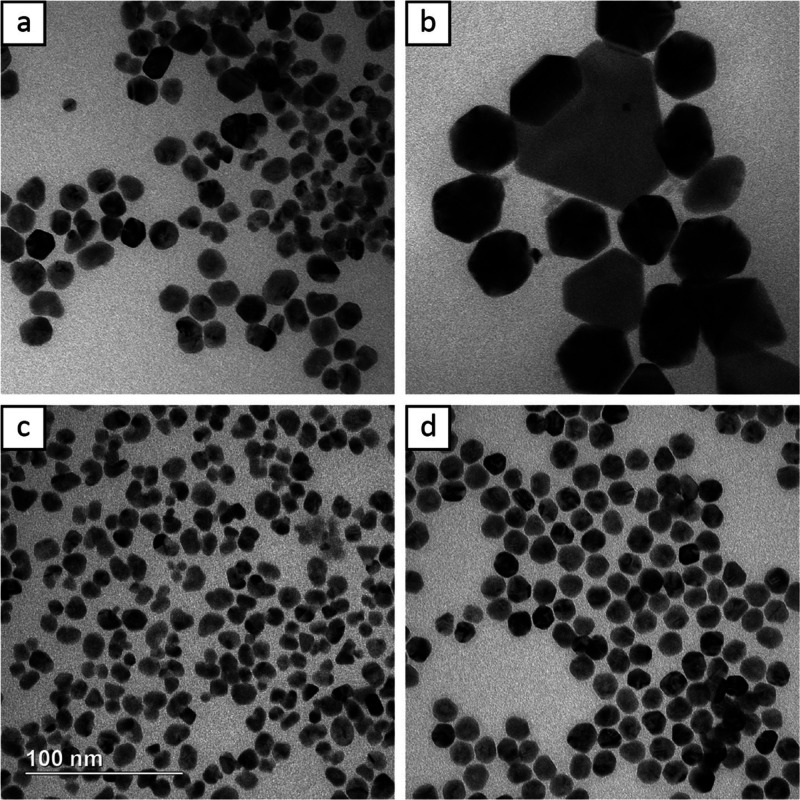
TEM images of Rh@Au nanocrystals synthesized with (a, b) varying amounts of PVP and (c, d) AA. Nanocrystals were synthesized with (a) 15 and (b) 20 mg of PVP, respectively. Nanocrystals synthesized with (c) 2.5 and (d) 10 mg of AA, respectively. The scale bar applies to all panels.

To fully understand the effects of the remaining synthesis components, the amounts of PVP and AA added were also varied (Figure 7). Increasing the amount of PVP to 15 and 20 mg first hindered and then completely inhibited Au deposition on the Rh seeds (Figure 7a,b). PVP is known to be a mild reducing agent, and while some increase in reducing power through excess PVP is possible, this effect is likely minimal under such low concentrations and mild reaction conditions.^33^ Instead, the inhibited deposition and increased self-nucleation are likely the result of PVP blocking the Rh surface. Although PVP might not have much direct influence on the reduction rate, blocking the surface can alter the kinetics of a surface-catalyzed reaction. The result was an increased concentration of free Au atoms and/or ions in solution, which eventually satisfied the conditions for self-nucleation. Varying the amount of AA used had a more nuanced effect. The highly irregular nanocrystals in Figure 7a include a mix of bowtie, kidney, and triple nucleation morphologies, indicating simultaneously weak and strong reduction. This counterintuitive mix is the result of decreasing the amount of AA to 2.5 mg without changing the amount of NaOH added. While the concentration of the reducing agent was decreased, the strength of the remaining AA was increased as the pH increased. The opposite effect was observed in Figure 7d. The decreased potency of AA meant that despite increasing the amount of AA, the reduction strength did not increase nearly as much. Instead, a somewhat gentle reduction was achieved with no increase in the number of nucleation sites.

**Figure 8:**
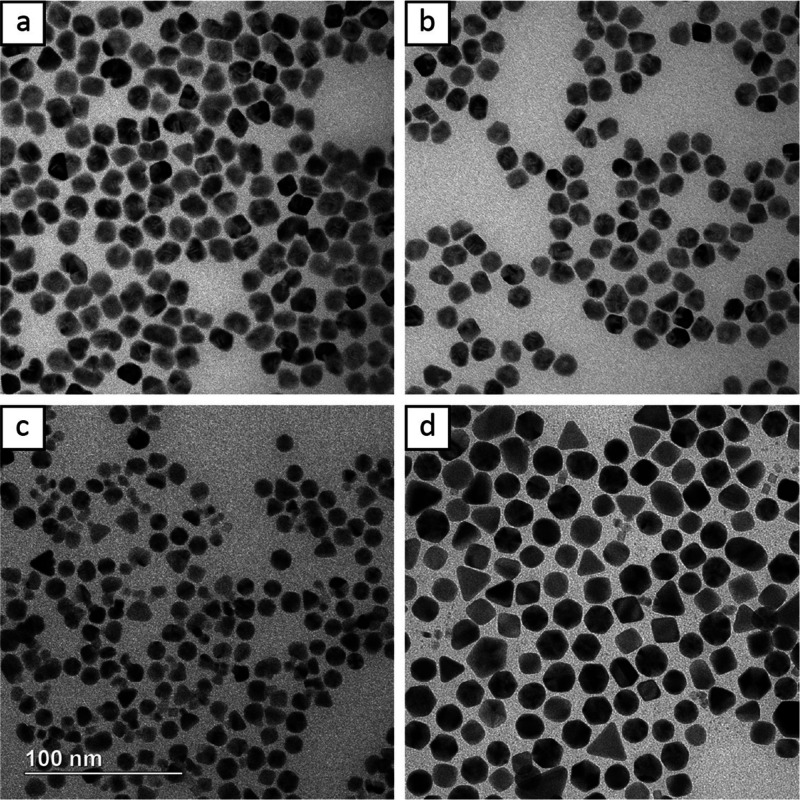
TEM images of Rh@Au nanocrystals synthesized at (a) 35, (b) 45, (c) 55, and (d) 75 °C. The scale bar applies to all panels.

Figure 8 shows TEM images of Rh@Au nanocrystals synthesized at different temperatures. Increasing the temperature by 10 °C to 35 °C does not appear to significantly influence the final particle morphology (Figure 8a), however, further increasing the temperature to 45 °C did lead to a more obvious increase in diffusion, reducing some shape distortion (Figure 8b). The relationship between temperature and reaction kinetics is nonlinear. Increasing the temperature another 10 °C past 45 °C to 55 °C increases the reducing power significantly, leading to notable self-nucleation. This is easily observed by the presence of uncoated Rh seeds and Au icosahedra in Figure 8c. Once the temperature was raised to 75 °C, no more Rh@Au core–shell nanocrystals could be formed (Figure 8d). Instead, a mix of Au nanocrystals including icosahedra, octahedra, and plates was observed alongside Rh seeds.

**Figure 9:**
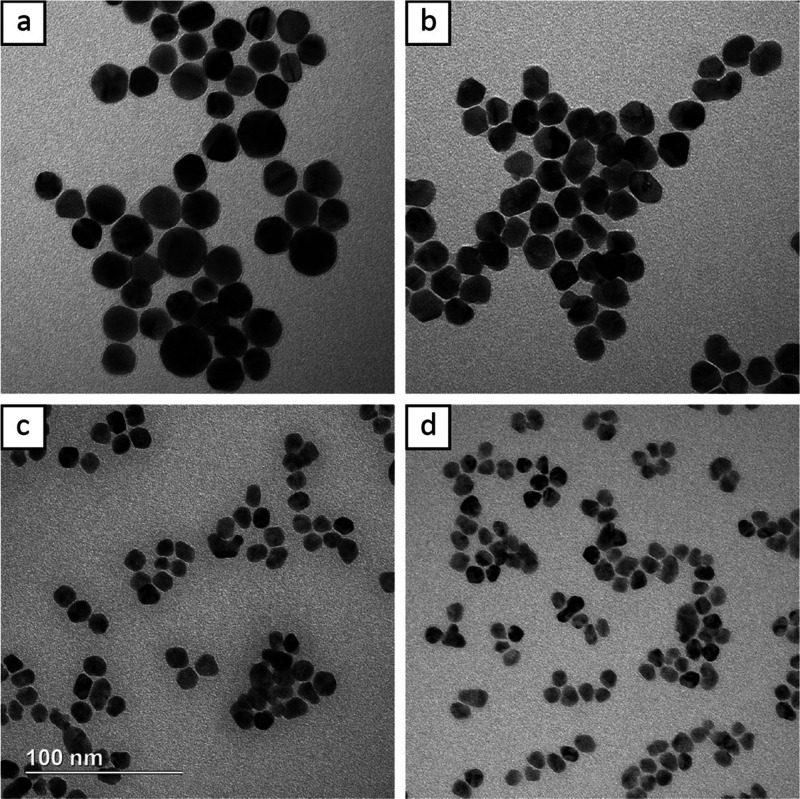
TEM images of Rh@Au nanocrystals synthesized using (a) 0, (b) 10, (c) 30, and (d) 50 μL of Rh seeds. The scale bar applies to all panels.

A facile strategy for tuning the size in core–shell nanocrystals is to change the number of seeds used in the protocol. Reducing the number of seeds effectively increases the amount of precursor available per seed, increasing the particle size. Figure 9 shows TEM images of the nanocrystals synthesized with 0, 10, 30, and 50 μL of Rh seeds. Predictably, using no Rh seeds produced Au icosahedra of various sizes (Figure 9a). Using 10 μL of the Rh seeds resulted in larger Rh@Au nanocrystals as might be predicted; however, the Rh cores were displaced from the center. As the proportion of Au(III) precursor to Rh seeds increased, so did the proportion of PVP. This led to partial surface obstruction on the Rh seeds and a much milder result than the one discussed in Figure 7a. Increasing the volume of seeds added to 30 and 50 μL (Figure 9c,d) produced smaller nanocrystals with morphologies similar to those observed when the volume of precursor added was decreased (Figure 3b,c).

## Conclusions

In this work, we have demonstrated the facile epitaxial overgrowth of Au on Rh cubic seeds, despite a lattice mismatch of 7.2% under compressive strain. Careful examination of the strain not only in the shell but also in the core revealed the significance of using small seeds. The 4.5 nm Rh seeds were able to effectively distribute the large strain over their entire volume to help prevent lattice defects in the Au shell. By carefully controlling the kinetic knobs with NaOH and KBr, we were also able to induce a unique two-site nucleation growth mechanism. This allowed the Rh core to be more centrally located in the Rh@Au truncated octahedra. The size of the Rh@Au truncated octahedra could be increased from 13.8 to 17.0 nm by adding more Au(III) precursor. While syntheses of highly lattice mismatched core–shell nanocrystals have long existed in the literature, the success of this synthesis offers insight into useful strategies for their rational design.

## Supporting Information Available

Additional TEM and STEM images of Rh seeds, EDX and line-scan of Rh@Au nanocrystals, XRD of Rh seeds and Rh@Au nanocrystals, UV–vis spectra of Rh@Au nanocrystals, and TEM images of Au nanocrystals (PDF)
