# Supplementary material for: Rh@Au Core–Shell Nanocrystals with the Core in Tensile Strain and the Shell in Compressive Strain
Source: J Phys Chem C Nanomater Interfaces. 2024 Jan 15;128(3):1377–85. doi: 10.1021/acs.jpcc.3c06793 (PMC10823532; doi:10.1021/acs.jpcc.3c06793)
Supplement: Supplementary file 1 — jp3c06793_si_001.pdf [file jp3c06793_si_001.pdf]

Supporting Information for

Rh@Au Core-Shell Nanocrystals with the Core in Tensile Strain and the Shell in Compressive Strain

Veronica D. Pawlik,[†] Annemieke Janssen,[†] Yong Ding,[¶] and Younan Xia*[†§]

[†]School of Chemistry and Biochemistry, Georgia Institute of Technology, Atlanta, GA 30332 United States

[¶]School of Material Science and Engineering, Georgia Institute of Technology, Atlanta, GA 30332 United States

[§] The Wallace H. Coulter Department of Biomedical Engineering, Georgia Institute of Technology and Emory University, Atlanta, GA 30332, United States

[*]Address correspondence to younan.xia@bme.gatech.edu

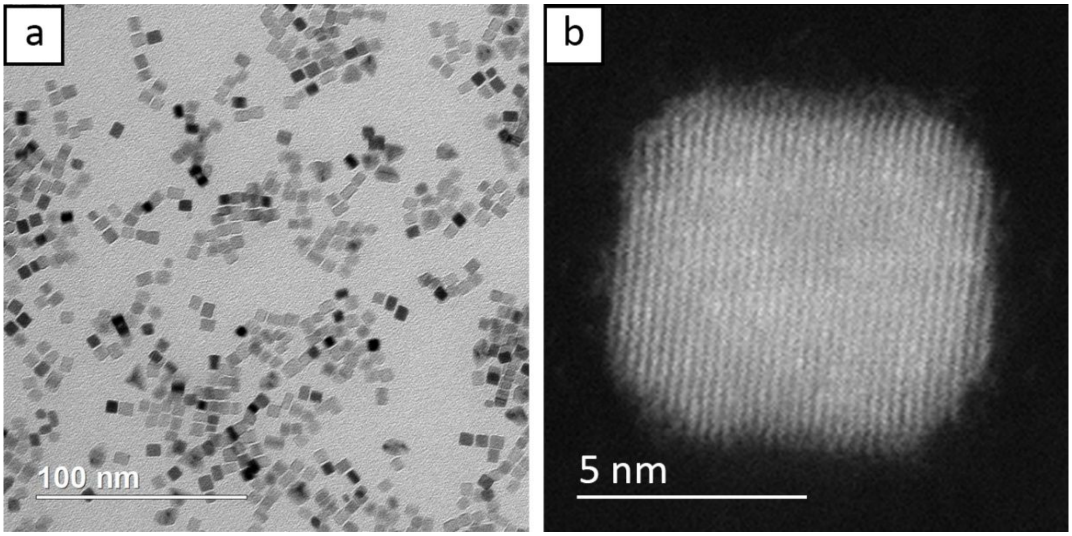

Figure S1. (a) TEM and (b) STEM images of the as-synthesized Rh nanocubes to be used as seeds in the present work.

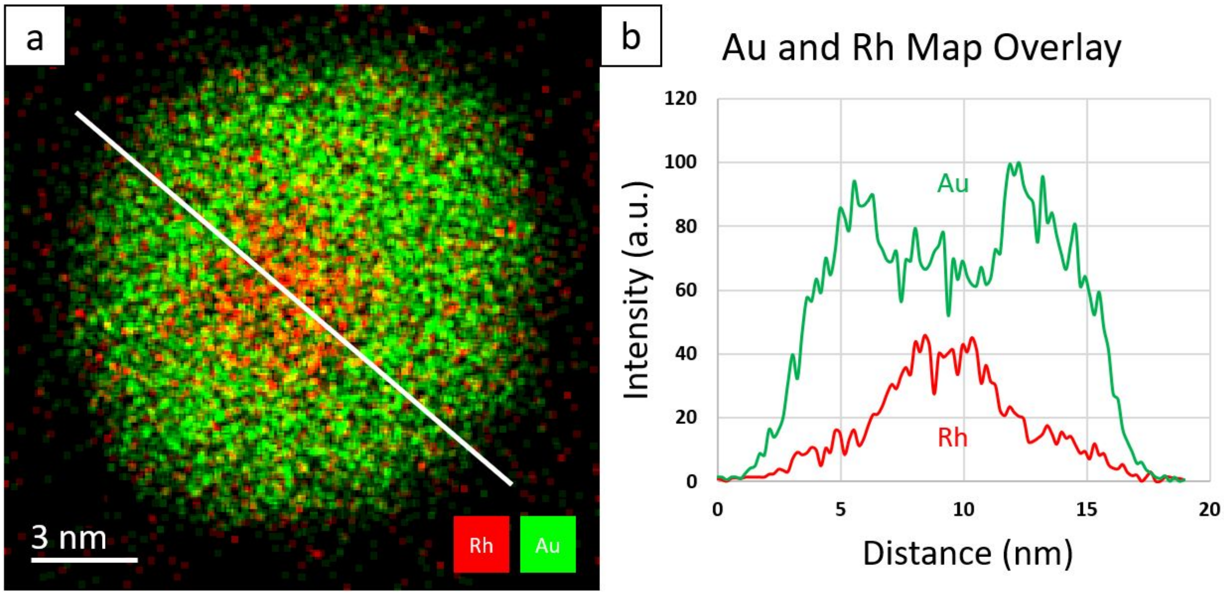

Figure S2. (a) EDX of an Rh@Au nanocrystal and (b) the corresponding line scan that shows the elemental distributions of Au (green) and Rh (red) along the white line in (a).

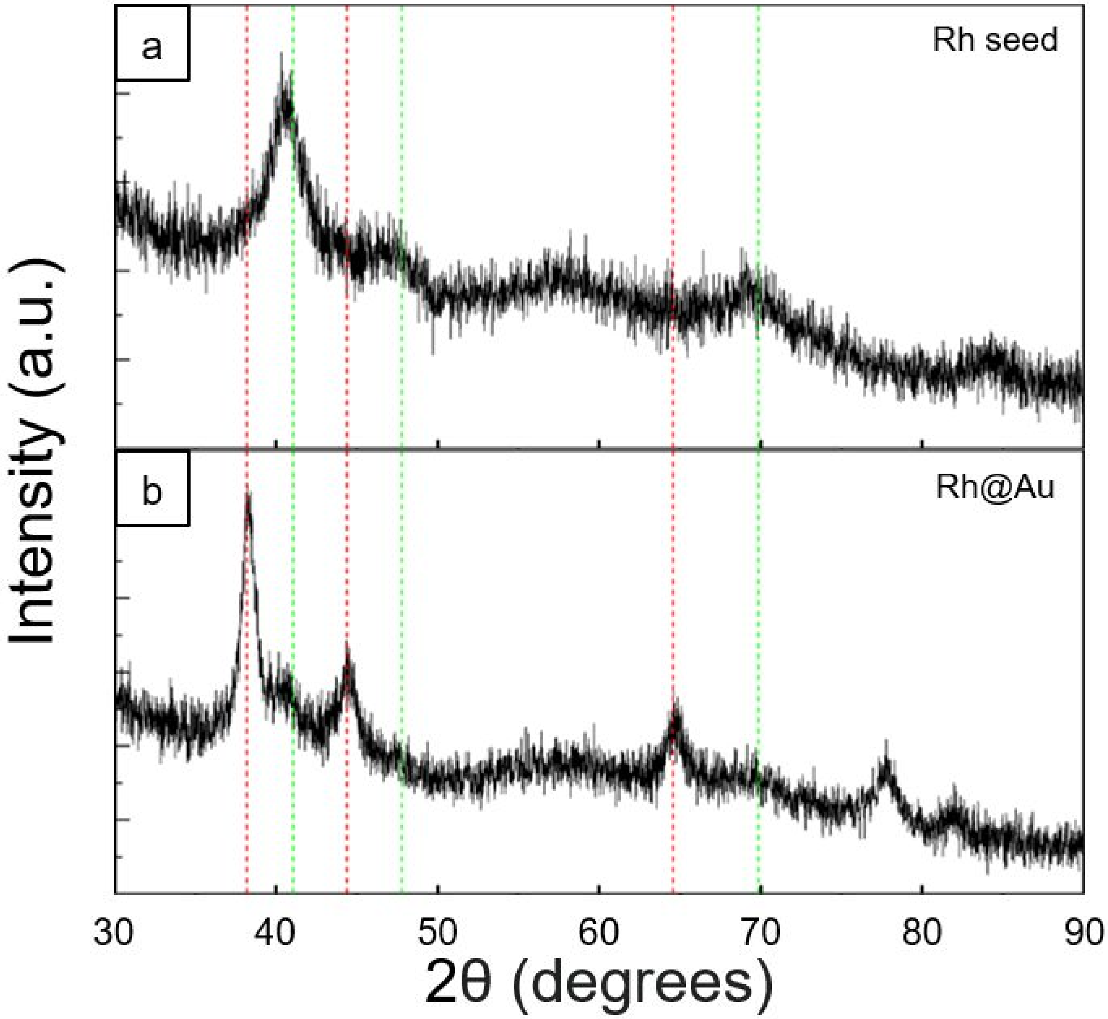

Figure S3. XRD patterns of (a) Rh seeds and (b) Rh@Au core-shell nanocrystals. The dashed lines mark the reference angle positions for bulk Au (red) and Rh (green).

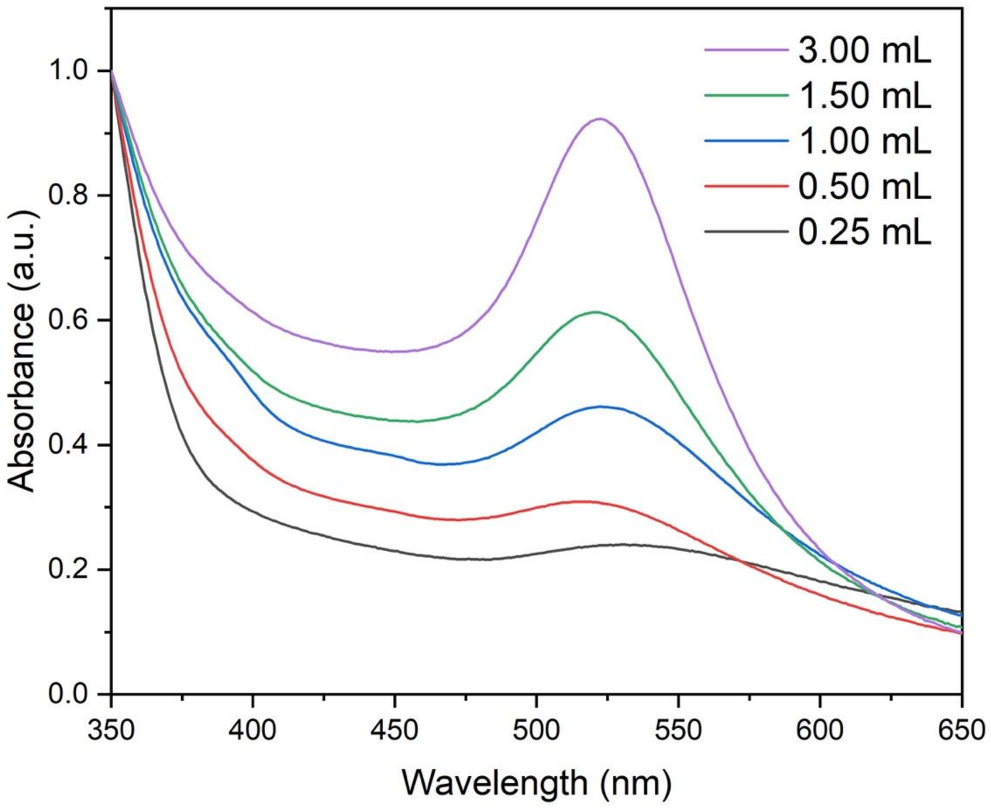

Figure S4. UV-vis spectra of the Rh@Au nanocrystals at different stages of growth corresponding to the TEM images in Figure 2.

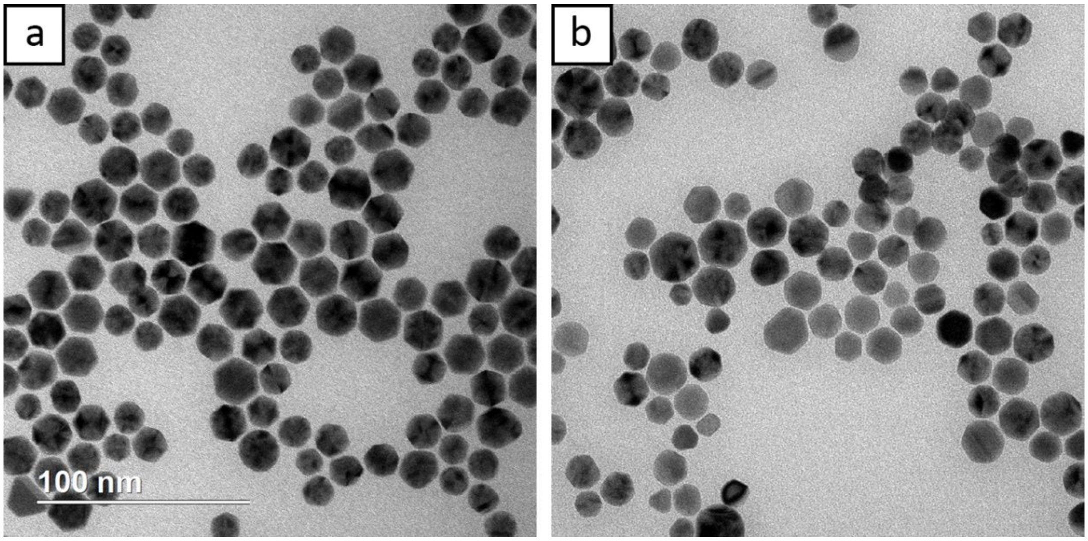

Figure S5. TEM images of the nanocrystals synthesized in the absence of Rh seeds using (a) one-shot injection and (b) an injection rate of 0.200 mL/h. The scale bar applies to both panels.
